# Feasibility study on using a detection dog to localize hibernacula of *Vipera berus*

**DOI:** 10.1038/s41598-026-51778-3

**Published:** 2026-05-09

**Authors:** Madita Schemel, Sascha Buchholz, Jelena Mausbach

**Affiliations:** 1 Institute of Landscape Ecology, University of Münster, Münster, Germany; 2 Centre for Integrative Biodiversity Research and Applied Ecology (CIBRA), University of Münster, Münster, Germany; 3 Artenspürhunde Schweiz, Olten, Switzerland

**Keywords:** reptiles, population monitoring, species conservation, wildlife detection dog, line-up, scent, Ecology, Ecology, Zoology

## Abstract

The European adder (*Vipera berus*), classified as critically endangered in Germany, faces severe threats from habitat loss, particularly the decline of primary habitats such as peatlands. Peatland restoration is essential but may pose risks when hibernacula are not considered during rewetting, potentially leading to flooding, site loss, and population declines. Similar risks arise from construction activities in secondary habitats. Protecting these key sites is therefore vital for species conservation but requires reliable methods to locate them. Traditional monitoring approaches, such as visual surveys and telemetry, are often invasive, imprecise, or resource-intensive. This study tested whether a trained adder detection dog could identify adder scent—using sheds and fecal swabs—beneath substrate in controlled line-up experiments and under field conditions. In the line-up tests, we assessed how sample type, burial depth, and substrate influenced detection performance, whereas field searches evaluated detection success under more realistic conditions. In a pilot study, we further examined whether the dog could generalize from collected odor samples to live adders by testing its ability to locate the hibernacula of two previously tagged individuals. In the line-up experiments, the dog identified both sheds and fecal swabs with high sensitivity, performing slightly better with sheds. Detection remained effective at depths of up to 1.2 m, although detection success declined with increasing depth. Under field conditions, the dog reliably detected samples buried at a depth of 50 cm without any false indications. In the pilot study, the dog successfully located hibernating adders, demonstrating that training non-invasively with collected odor samples rather than live animals can be sufficient for this purpose. These findings suggest that detection dogs represent a promising, non-invasive, and efficient tool for locating adder hibernacula. Future studies should include multiple dogs, larger sample sizes, and additional tagged individuals to further validate detection performance under field conditions.

**Supplementary Information:**

The online version contains supplementary material available at 10.1038/s41598-026-51778-3.

## Introduction

Global biodiversity is declining rapidly, with 19.4% of species in Europe at risk of extinction, including 18% of vertebrates^1^. This loss is primarily driven by land-use change and climate change^1^. Reptiles are particularly affected, with 21.1% (1,829 species) of the nearly 10,000 known species currently considered threatened worldwide^2^. This development even affects reptile species with formerly wide distributions, such as the European adder (*Vipera berus*)^3^. Across large parts of Europe, particularly in Western Europe, the species has experienced pronounced population declines^4–6^. In Germany, adder populations have decreased by an estimated 50–70% over the past 100 years; the species is therefore classified as highly endangered nationwide and, in some federal states including North Rhine-Westphalia, as threatened with extinction, with continuing negative population trends^4,7^. As a predator, it also fulfills a functional role within ecosystems, meaning that population declines may have ecological consequences beyond the species itself^3^. The main drivers of this decline are habitat loss and increasing habitat fragmentation^7,8^. Due to its limited dispersal ability and high site fidelity, the adder is particularly sensitive to habitat alteration, as habitat fragmentation rapidly leads to small, isolated populations with an increased risk of inbreeding and local extinctions^3,4^. This highlights the importance of conserving existing habitats and structures, especially peatlands, which constitute key habitats for adders but have been severely reduced and degraded over recent decades^7,9,10^. While peatland restoration is an essential conservation measure, it also entails risks^7,11^. Inappropriate renaturation practices, particularly uncontrolled increases in water levels, can destroy critical hibernacula, which are often used repeatedly by adders, leading to high mortality and local population collapses^7,11,12^. Similar risks also arise in secondary habitats, for example during construction or habitat management activities^7^. Adders hibernate in protected sites such as root stumps, embankments, and burrows, with depths ranging from 5 cm to 1.25 m^7,10^. They are very reluctant to use newly created hibernacula, making preservation of existing ones essential^11^. Traditional methods for locating hibernacula are often inefficient and time-consuming, as they mainly rely on rough estimates from basking site observations and are therefore imprecise^7,10,12^. Telemetric surveys allow accurate localization but involve relatively high costs, cause significant disturbance to the animals, and risk missing untagged individuals using different hibernacula. Non-invasive, cost-effective methods are needed for more efficient hibernacula detection. One solution could be the use of wildlife detection dogs. Studies show that dogs outperform humans in 88.7% of cases, detecting targets faster and more accurately, even in dense vegetation^13–15^. While mammals and birds are up to now relatively well studied using detection dogs, reptiles remain underrepresented^14^. Even though some research has demonstrated their ability to detect snakes, lizards, or tortoises^15–17^, studies focusing on reptiles occupying underground refuges are still scarce. Existing work on tortoises and Indigosnakes in burrows has shown promising results^15,18^, yet it remains largely unclear how detectability is affected by burial depth and substrate type. Environmental factors such as wind, temperature, and humidity may further influence detection success^19,20^. Although dogs have been shown to detect amphibians beneath a 20 cm soil layer^21^, the applicability of this approach to deeper underground reptile refuges has not been evaluated. As a result, it remains unclear whether this approach also works for reptiles that are in deeper hibernation, such as the European adder.

This study evaluates the feasibility of using detection dogs to locate adder hibernacula by assessing odor detectability under controlled conditions and detection performance in the field. Specifically, it tests whether a trained adder detection dog can reliably detect adder odor in standardized line-up tests across varying depths and substrates, and examines how sample type, substrate type, and depth affect detection success. The study further evaluates odor detection beneath substrate under different field conditions and assesses whether the dog can generalize from training samples to live adders and accurately locate them in the field.

## Materials and methods

The study was conducted in three experimental steps. These comprised controlled depth-variable line-up trials, a field experiment with double-blind searches with buried odor samples at two sites, and a pilot field experiment conducted in areas with known adder hibernation. Adder sheds and fecal swabs served as odor samples in the line-up and field experiments.

### Samples and storage

Adder sheds were collected from both wild and captive individuals using disposable gloves to avoid contamination. All sheds were stored individually in sterile, tightly sealed glass jars. To prevent mould growth, sheds were allowed to dry completely prior to storage. Fecal samples were collected using sterile swabs during routine monitoring at the Dortmund–Ems Canal, when snakes produced fresh scat during handling. Swab samples were transported in cooled containers and stored at − 18 °C. Prior to each trial, swabs were defrosted for approximately 30 min. In total, 21 sheds and 45 fecal swabs were used across training and experimental trials. Fecal swabs were obtained from approximately seven different individual adders, resulting in a total of 45 swab samples. Sheds were collected from 21 individuals, including nine samples from three wild adder populations in Germany and twelve from individuals housed in outdoor enclosures at various zoological institutions in Germany. Before use, all sheds were visually examined and confirmed as *Vipera berus* based on characteristic morphological features visible in the shed skin. The individuals providing fecal swabs and sheds did not overlap. Samples used during training were not reused in subsequent experimental trials.

### Detection dog selection and training

A 23-month-old male English Springer Spaniel was selected for its proven scent detection abilities. Training began at 16 weeks of age and lasted for approximately 1.5 years prior to the start of the experiments. Initial training used chamomile tea as a substitute odor before transitioning to adder scents. The dog was trained using classical and operant conditioning to indicate a target odor by lying down. Correct indications were marked with a clicker and immediately followed by delivery of a primary reward (food or play). A clicker functions as a conditioned (secondary) reinforcer that marks the precise moment of the correct response and bridges the temporal delay to reinforcement^22^. Training comprised searches in a standard line-up of up to ten clay pots, as well as multiple searches using a depth-adjustable line-up that was also employed in the experiments of this study (Supplementary Fig. 1). Both training approaches were conducted using the substitute odor and the target odors (adder sheds and fecal swabs). Field search training was carried out across multiple areas differing in habitat type and structural complexity, including grasslands and pastures, forested sites, and rock piles. Training scenarios included variation in target placement, pooled and blank searches and included single-blind as well as double-blind search designs. Searches at varying depths were conducted with both the substitute odor and the target odors. Both sample types were used equally throughout training to prevent bias toward a specific odor source.

### General experimental procedures

Line-up and field experiments were conducted using a double-blind design. In double-blind searches, the person who placed the samples or who knew the position of the search object was not present during the search to prevent potential handler bias and unintentional cueing^23,24^. For all experiments, the dog was handled on a 5 m leash. Latex-free, powder-free disposable gloves from different manufacturers were used for sample handling to minimize contamination. Odor samples were placed inside plastic tubes (fecal swabs: 1 cm × 3 cm; sheds: 7 cm × 2.5 cm), which served as carrier material to allow sample reuse while reducing odor contamination of the experimental setup. To enable odor release, tube openings were covered with fiberglass mesh rather than lids. Unused sterile swabs placed in identically prepared tubes were used as blanks in swab trials. In experiments using sheds, identically prepared tubes without odor material served as blanks. After initial use, swab samples were stored at 5 °C until reuse. For the line-up and field experiments, separate sample sets were used. One batch of swabs and sheds was assigned exclusively to the line-up experiments, and another batch exclusively to the field experiments. Within each batch, samples were randomly selected for individual trials. In field searches, separate sample sets were used for each site to ensure that swab samples from the embankment and the pasture were not mixed. This approach ensured consistent sample quality and prevented potential degradation caused by prior burial or prolonged exposure to ambient temperatures. If a sample used in the line-up experiments became heavily soiled and was considered unsuitable for further use (e.g., due to visible contamination with peat), it was removed from the batch and replaced to maintain a constant number of samples within the experimental set.

### Depth-variable line-up

To assess the effects of sample type, depth, and substrate, a depth-adjustable line-up was used (Supplementary Fig. 1). The setup included five PVC pipes in a box (120 cm high × 40 cm wide × 260 cm long), each run filled with either stones (3–10 cm) or white peat—substrates typical of adder hibernacula. Each test included one positive and four negative targets with its position chosen randomly, with 35 runs per sample/substrate type (140 runs total). Hiding depth increased by 20 cm every five runs, from surface level to 120 cm. The sample tubes were positioned such that the fiberglass side faced upward or at least diagonally upward, and never downward, to ensure that upward odor dispersal was not artificially constrained. Used substrate was replaced after each run to avoid scent contamination. Stones that had already been used once were placed in water overnight, thoroughly rinsed and air-dried afterwards. This allowed the stones to be reused the next day. Samples were exposed for at least 30 min per 20 cm of depth (up to 180 min), following Glover et al.^21^. The dog was given up to two opportunities to search all tubes. If an alert occurred during the first search round, the trial was terminated, and no second round was conducted. Misses and false alerts were recorded if no correct indication was made by communicating with the person that placed the samples by radio after the dog showed the indication behavior. Correct indications scored as 1; all others as 0.

### Field experiment

To evaluate detection success under realistic field conditions, transect searches were conducted. Searches took place on a horse pasture in Soest (North Rhine-Westphalia, Germany) and on a north-facing embankment at the Dortmund–Ems Canal near Senden (North Rhine-Westphalia, Germany); both sites were known to be free of adder presence. At each site, twelve unique transects of approximately 50 m² were established to prevent interference from relict odors. Square transects were used on the pasture, whereas elongated transects were used on the embankment to account for the linear structure and variable width of the site (mean dimensions approximately 3.5 × 15 m). All transects were searched once, with an equal number of transects per sample type. Searches were conducted between January 27 and February 10, 2024, across five days; on three days, searches were split into morning and afternoon blocks, whereas the remaining two days consisted of a single block each. Per transect, 2–4 positive samples and four blank samples were buried at approximately 50 cm depth, a depth representative of typical adder hibernacula in western Europe^10^, resulting in a total of 70 positive samples (35 per site). The placement of positive and blank samples followed no predefined pattern. After burial, the soil was compacted to avoid visual cues indicating sample locations. To prevent the dog from tracking the assistant’s scent, the assistant moved through each transect in a criss-cross pattern and deliberately touched different areas throughout the transect. In some locations, the soil was disturbed without placing a sample. Samples were buried approximately 75 min prior to the start of each search, following Glover et al.^21^. Searches were initiated by a verbal command from the handler, who was free to choose the starting position. For each alert, the time required until the indication was recorded; following documentation of the alert, the search was resumed, and time measurement was restarted. Wind speed and direction, temperature, and humidity were recorded. Upon an alert by the dog (e.g. lying down), time was stopped and GPS coordinates and photographic documentation were collected, after which the dog resumed searching. Once the handler considered the transect fully searched, the search was terminated and the next transect was visited. Detection points were subsequently compared with the actual sample locations, and correct, false, and missed indications were documented after completion of the search. All samples were removed after each transect search. To prevent frustration, the dog was rewarded after each alert.

### Feasibility pilot field test with live adders

The surveys were conducted in the Venner Moor near Senden (Coesfeld district, North Rhine-Westphalia, Germany) and in a second study area located along the nearby south-facing side of the Dortmund–Ems Canal; both areas are known to support relatively large adder populations. The Dortmund–Ems Canal study area comprised a south-facing stone embankment of approximately 2 km in length, adjacent wooded areas, and three substitute habitats consisting of stone bars and brushwood piles. The tests took place on January 10, 2024, under dry weather conditions, at a temperature of − 4 °C and with wind speeds of up to 6.2 m/s. In total, four transect searches were conducted. At each study area, one search was carried out in a transect with a telemetrically confirmed hibernaculum (based on telemetric monitoring data from November 2023) and one search was conducted in a transect with a high probability of containing a hibernaculum, as assessed by expert judgement. In areas with telemetrically confirmed hibernacula, the handler was assigned transects of approximately 375 m² (15 × 25 m) at the Dortmund–Ems Canal and approximately 320 m² (8 × 40 m) in the Venner Moor. The two potential hibernacula areas comprised transects of approximately 70 m² each. The confirmed transects were located in the eastern replacement habitat near the Dortmund–Ems Canal and on a peat dam in the Venner Moor. To ensure a double-blind search, the person who was aware of the telemetrically determined adder locations remained out of sight during the searches. Upon an alert, the dog was initially rewarded with a click and a ball, after which the position was checked. All alerts, including those without immediate verification, were rewarded, as the presence of additional, non-tracked individuals could not be excluded. Search data, including position, weather parameters (temperature, wind speed, wind direction, relative humidity, precipitation) and time, were recorded.

### Statistical analysis

We calculated sensitivity as a classical signal detection theory parameter using the formula (correct alerts / positive targets) × 100. In addition to sensitivity, effectiveness was calculated as (correct alerts / [positive targets + false alerts]) × 100 as a complementary measure of detection performance that accounts for both missed targets and false alerts following Matthew et al.^25^. This prevents an overestimation of performance, for example in dogs that indicate frequently but non-specifically. Both metrics were calculated for overall performance as well as separately for the different combinations of sample type and substrate.

Analyses were conducted in RStudio v2023.12.0^26^ using R statistical software v4.3.2^27^. The effects of sample type, depth, and substrate on search success (correct = 1, missed/false = 0) in the depth line-up trials were analyzed using a binomial generalized linear model (GLM). Model selection was based on the lowest AIC, and overall model significance was assessed using likelihood-ratio (omnibus) chi-square tests comparing the full model to the null model. Effect sizes and model fit were evaluated using odds ratios and Nagelkerke’s pseudo-R², while model assumptions were examined based on deviance residuals and simulation-based residual diagnostics implemented in DHARMa^28^. Models were tested for overdispersion, multicollinearity, and residual autocorrelation. Multicollinearity was evaluated using variance inflation factors (VIFs), and residual autocorrelation was assessed using model-based diagnostics implemented in the R package performance^29^. To examine whether the effect of depth depends on substrate type and sample type, we considered models including interaction terms. However, estimating such interactions requires reliable model fitting and sufficient variation within each substrate–sample type combination to estimate separate depth effects. In our data, correct and incorrect detections were highly unbalanced within most combinations, such that interaction models could not be fitted reliably and would not yield trustworthy results.

Due to quasi-complete separation of the data (69 correct detections out of 70), inferential statistical analyses of detection success were not conducted. Under such conditions, parameter estimates from generalized linear or mixed-effects models are unstable and not meaningful, regardless of model structure. Detection performance is therefore reported descriptively using sensitivity and effectiveness metrics. Inferential analyses of search time were likewise not performed, as environmental conditions were strongly structured by site and time block and did not show sufficient variation to allow reliable estimation of individual effects. Search time was therefore reported descriptively to provide an overview of the dog’s search speed at both study sites. No statistical analysis was performed for the pilot study due to limited adder sightings. All plots were generated using ggplot2 and ggpubr. Analyses were conducted using the R packages tidyverse^30^, car and psych^31^.

### Ethics declaration

This study was conducted in full compliance with ethical standards for wildlife research and in accordance with German and European Union regulations concerning the protection of native species. All research activities were approved by the appropriate regulatory authorities, including the Lower Nature Conservation Authority, Coesfeld District and corresponding landowners. The European adder is a protected species in Germany; therefore, all work involving capture, handling, or monitoring was carried out with minimal disturbance to the animals and their natural habitat. Trained personnel handled adders using safe and humane techniques to reduce stress and prevent injury to both animals and researchers. No animals were harmed, killed, or removed from their habitat unless specifically authorized under the issued permit. After data collection, all individuals were released at the site of capture as promptly as possible. The study design followed the ethical guidelines of the German Animal Welfare Act (Tierschutzgesetz) and the European Directive 2010/63/EU on the protection of animals used for scientific purposes, as applicable to non-invasive field studies. All data collection procedures prioritized the conservation and welfare of *Vipera berus* and aimed to contribute to the species’ long-term protection and understanding.

## Results

### Depth-variable line-up

**Fig. 1:**
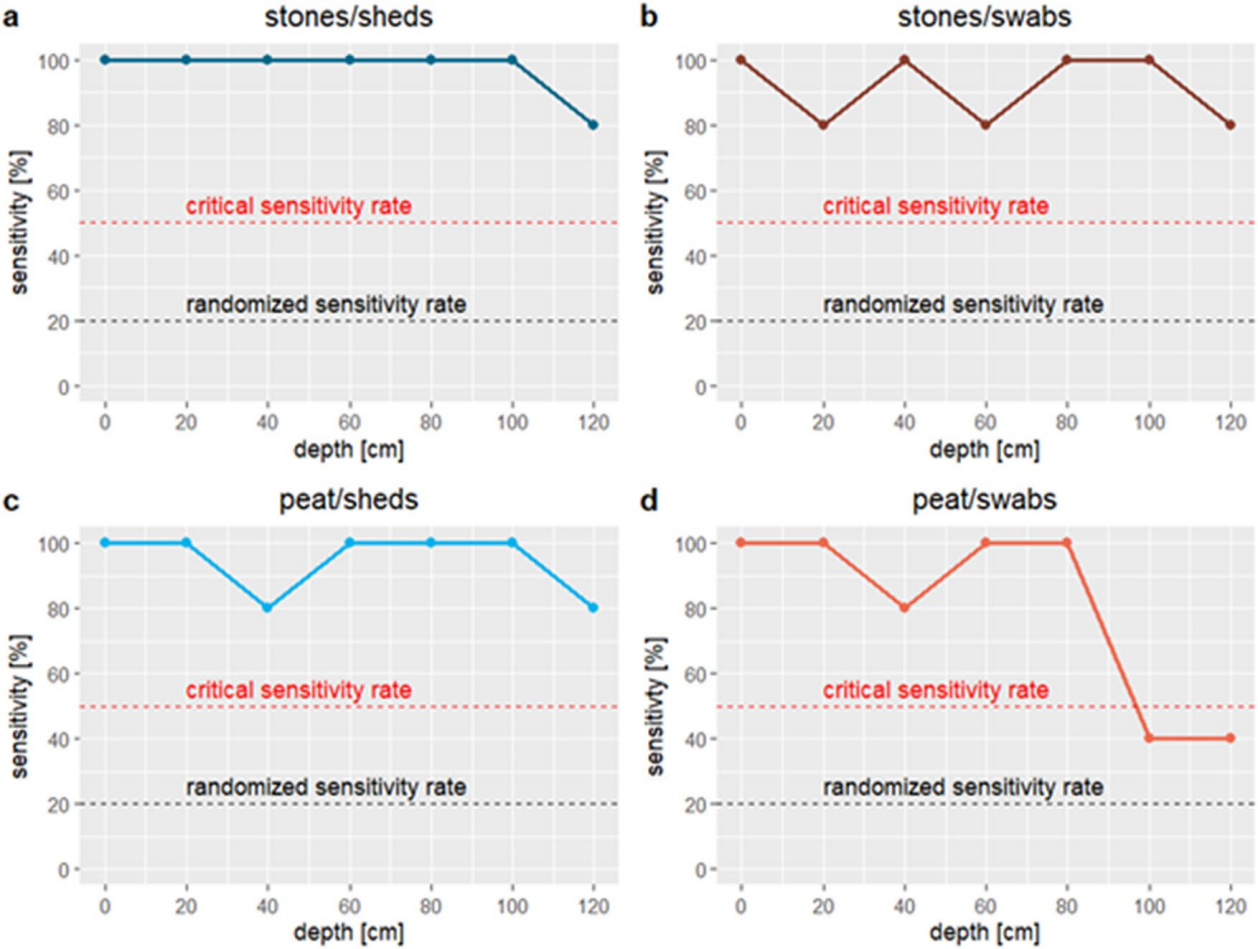
Change in sensitivity [%] from 0 to 120 cm depth depending on treatment (**a**: stones/sheds; **b**: stones/swabs; **c**: peat/sheds; **d**: peat/swabs).

Of 140 positive targets, the dog correctly identified 127, missing 13 and producing one false alert at a negative target in seven runs (Supplementary Table 1). This results in an overall sensitivity of 90.71% and effectiveness of 86.39%, both well above chance levels (Supplementary Table 1). Sensitivity remained above 50% in all but two cases (Fig. 1). The GLM significantly outperformed the null model (Table 1; *P* < 0.001), showing the dog was more accurate with shed samples than swabs (*P* = 0.043). Sensitivity and effectiveness were higher for sheds (95.7% and 93.1%) than swabs (85.7% and 77.9%). Substrate type had no significant effect (*P* = 0.13).

**Table 1:** Test statistics of the binomial GLM: response variable = search success/ correct indication (0/1), predictor = sample type, substrate type, depth [cm])

Omnibus-Test (χ²)	Pseudo-*R*²	AIC	predictor	Estimate	SE	*P*-value	odds ratio
0.000998***	0.2379	78.278	Sample Type	-1.44295	0.7128	0.04295*	0.2362303 (sheds > swabs)
			Substrate	1.00325	0.6652	0.13153	0.3666862 (stones > peat)
			Depth [cm]	-0.02644	0.0096	0.00621**	0.9739044

Depth had a significant impact on correct indications (Table 1, GLM, *p* = 0.00621). With increasing depth, sensitivity declined, while missed and false detections increased, especially in deeper depths (Fig. 1; Supplementary Table 1). Few false alerts occurred, even at 20 cm depth. Sensitivity remained above 80% in most tests but dropped sharply to 70% at 120 cm depth, while effectiveness decreased to 63.6%. (Supplementary Table 1).

In peat–swab trials, sensitivity dropped from 100% to 40% at 100 cm depth, with three missed alerts recorded across five runs at 100 and 120 cm. In one of the three 100 cm trials with missed alerts, the dog gave no indication, while the remaining two included a false alert. Effectiveness also declined steeply beyond 100 cm, dropping to 28.6%, with a slight increase to 40% at 120 cm, representing a 71.4% reduction compared to 80 cm depth (Supplementary Table 2).

### Field tests

Of the 70 positive samples, the dog correctly identified 69, including 35 on the pasture and 34 on the embankment. The single missed swab occurred due to a search that had to be terminated following an external disturbance. No false alerts were recorded, and blank samples were consistently ignored. Sensitivity and effectiveness reached 100% on the pasture and 97.1% on the embankment. Detection performance was slightly lower for fecal swabs (97.1%) than for sheds (100%), resulting in an overall sensitivity and effectiveness of 98.6%. The mean search time per positive sample was 37.5 s (SE = 3.18), with an average of 35.17 s on the pasture (median = 28 s) and 38.97 s on the embankment (median = 32 s) (Supplementary Fig. 2).

### Feasibility pilot test

During pilot transect searches at the canal and in the Venner Moor, the dog accurately located the position of the previously tagged adders in their hibernacula, with indications within 50 cm of telemetry points. In the first canal search, the dog alerted twice (after 60 and 80 s) at a stone bar, with the second matching the hibernaculum location. In the Venner Moor, the dog indicated after 57 s on a peat moss bump, also matching the known hibernaculum site. In two control searches without tagged adders, the dog still indicated likely hibernaculum sites. The first alert (after 40 s) was at a stone bar; the second (after 130 s) was near a tree stump, where a potential burrow was later found.

## Discussion

Our results highlight the dog’s high detection accuracy and efficiency, especially in identifying shed samples, and underline the importance of considering depth when planning hibernacula surveys. Additionally, the dog’s success in both controlled and field searches suggests its potential for broader use in conservation efforts, particularly for species like adders in challenging environments, even though we are aware that general conclusions due to a single tested dog are limited.

### Depth-variable line-up

This study demonstrated that a specially trained detection dog could detect adder odor from both sheds and fecal swabs across various substrates in a depth-variable line-up. The high sensitivity and effectiveness (around 90%) achieved in these tests are comparable to other reptile line-up studies, such as those with tuatara, geckos, and green anole^17,32^. However, these studies are not fully comparable, as they did not use depth-adjustable line-ups, and the odor concentration remained constant in those studies, unlike the decreasing concentration in this study. A line-up study with increasingly diluted skin swabs from American bullfrogs found lower sensitivity and effectiveness, ranging from 49% to 87%^25^. Unlike the gecko study, the sensitivity for swabs was lower than for sheds, suggesting that fewer volatile organic compounds (VOCs) are released from swabs, thus reducing the detectable odor. This may be due to the larger sample size in the case of sheds, or the longer storage time of around four months, which could reduce the odor intensity, as observed in the American bullfrog study, where sensitivity dropped from 79% to 54% after four months^25^. Fresher swabs might yield better results, though it should be noted that the frog study used skin swabs, not fecal swabs. The relatively high standard error in this study also indicates some uncertainty in the results. The dog’s ability to detect adder odor under stones and peat at depths of up to 120 cm suggests that a trained dog could potentially locate adders in their hibernacula at the required depth and under typical substrate conditions^7^. However, detection performance significantly declined with increasing depth, a pattern that was also clearly visible in the course of sensitivity and effectiveness, particularly in the peat/swab tests. This decline in detection may be due to the difficulty of odor molecules diffusing through the substrate into the atmosphere, where they are detected by the dog. Although no significant effect of substrate type was detected in this study, it is reasonable to assume that VOC transport is facilitated in stone substrates via cracks and gaps, whereas in peat the movement of odor molecules through the soil matrix is slower and therefore likely requires more time for VOCs to reach the surface, particularly at very low concentrations. However, a real adder staying in the hibernacula for longer time than the odor samples tested in this study could potentially release more odor and smell more intense, making it potentially easier for the dog to detect even at higher depth. Factors like temperature, substrate affinity, and evaporation rates influence odor strength, while diffusivity is also affected by soil properties, moisture content, and bulk density^33,34^. As substrate thickness increases, the concentration of VOCs decreases, making detection harder. The sensitivity drops in peat and swab tests at depths over one meter, along with increased missed alerts, suggest, that insufficient VOC concentration passed through the peat to be detected. In contrast, sheds still had a higher detection rate at deeper depths, indicating that the VOC concentration was more detectable. False alerts may also occur due to residual odors from previous trials^35^. At greater depths, the VOC concentration of the positive sample might be comparable to residual odors, increasing the likelihood of false positives. In summary, the depth-variable line-up tests show that a dog can detect both odor types and highlight the influence of sample type and depth on detection. However, these laboratory findings may not fully translate to field conditions. The dog’s performance can be influenced by the test setup, and even in double-blind tests, handlers may unintentionally cue the dog by knowing that there is one positive sample^24,36^. Environmental factors such as temperature, wind speed, humidity, and terrain characteristics could also affect the dog’s performance in real-world searches^19–21,37,38^. Additionally, the PVC pipes used in the experiment may have optimised upward odor movement through a chimney effect^38^, thereby potentially enhancing odor availability compared to natural conditions. However, many adder hibernacula are naturally canal-like, such as vole burrows or gaps between stones, which could optimize odor dispersal for the dogs capability to detect it^10^. Also, dogs may differ individually for the maximum depth up to which they can detect odor, therefore a general conclusion regarding the mean maximum depth dogs can detect these samples needs to be tested with several dogs in future studies. As laboratory results may not always apply to field conditions, further outdoor testing is necessary^17^.

### Field searches

Field searches achieved nearly 100% sensitivity and effectiveness, indicating reliable detection of both odor types at 50 cm depth under varying environmental conditions. Slightly better results than in line-up tests may reflect increased motivation due to the more engaging field setting, reducing errors linked to repetition and distraction^39^. Additionally, relict odors were avoided since transects were only used once. However, detection rates may decline with greater depth, depending on substrate, even though deeper hibernacula are unlikely in the embankment due to groundwater presence at 50–70 cm, as adders require dry hibernation sites^7,10^. Detection success and time were similar at both sites. While substrate effects remain poorly understood, results suggest detection is feasible in both clay loam and stone substrates. Previous studies report mixed outcomes: Human Remains Detection Dogs performed better in sandy soil, while a newt dog was more successful in clay, attributed to porosity differences and moisture content^21,33^. Peat, although more porous than loam clay, may impair diffusion due to high water content^21,40,41^. The effects of weather-related variables on detection performance could not be formally evaluated, as environmental conditions varied only within a narrow range during the experiments, precluding robust statistical modelling. Temperatures were generally moderate, which may have been beneficial not only for detection performance but also for dog welfare, as high temperatures can cause fatigue and increased panting, potentially impairing olfactory performance, while low temperatures may reduce odor release^19,42^. Similarly, the influence of wind speed on detection performance could not be statistically assessed due to insufficient variation in wind conditions. Although wind is known to affect odor dispersion, its potential effects may have been further reduced by the experimental design, including short transects, searches conducted on a long leash following a systematic search pattern, and odor retention in moist substrates, which can limit odor movement and reduce the relevance of wind effects^19,43^. No big difference was found in detection between sample types, unlike in line-up tests. The dog was only marginally slower on the embankment but exerted more effort navigating slippery stones, highlighting the importance of adjusting transect size to prevent fatigue, especially in older dogs^17,39^. Due to uneven terrain, the dog sometimes gave alternative alerts (e.g. sitting instead of lying down), stressing the need for handlers to correctly interpret subtle behavioral changes^39,42,44^. One missed alert occurred due to interference by an off-leash dog, underlining the importance of boldness and focus in detection dogs and public awareness about not disturbing working dogs^39^. The double-blind setup ensured reliable results, as the dog ignored all blank samples. This suggests detection was not influenced by human or carrier odors, a common issue in similar studies^23,33,45^. Future studies should aim to eliminate plastic tubes to avoid compound odor cues and mimic natural odor dispersal better (channel effect by tubes). Few comparable studies exist on subterranean animal odor detection. One involving great crested newts buried at 20 cm reached 88% success rate^21^. In another, dogs detected western indigo snake sheds and live animals both above and below ground, though odors dispersed through open burrows rather than closed substrate^18,46^. Our study extends this by confirming detection through largely closed substrate lacking any major openings. Our sensitivity exceeds the 75% threshold set for certified desert tortoise detection dogs^43^ confirming high perforance. Overall, results support that a dog can detect here tested reptile odors under mostly natural conditions with comparable accuracy to that for human remains or amphibians tested so far. In a future study, ideally, this should be tested without tubes and by testing several dogs. These findings have implications for locating other cryptic species such as smooth snakes. However, previous studies have shown that each species should be tested individually for its olfactory detectability by dogs, as some may have evolved chemical crypsis as an anti-predator and hunting strategy^47^.

### Pilot study

Since odor samples represent only part of an animal’s scent, it was essential to test whether the adder detection dog could generalize this odor to living adders. The results of the pilot study suggest that such generalization is possible, as the dog successfully detected adders in natural hibernacula, indicating that trained odor cues remain detectable under natural conditions. However, of the two telemetrically confirmed adder locations, only the search in the Venner Moor can be considered an independent and valid alert. Although the dog also indicated the correct site at the Dortmund-Ems Canal during a double-blind search, the handler unintentionally directed the dog toward the location. This influence likely stemmed from prior survey knowledge and expectations held by the handler, which should be avoided in the future to reduce bias^42^. Dogs are highly responsive to subtle human cues due to their advanced social cognition^33,48,49^ and handler expectations can unintentionally lead to false alerts^24^. In contrast, during the Venner Moor search, neither the handler nor assistants had prior information. The dog searched independently and located the previously tagged adder’s hibernaculum without influence from human cues. The two alerts in transects without tagged adders suggest the likely presence of natural hibernacula. In the Venner Moor, one alert occurred near a tree stump on a peat dam. Nearby, a hole led into a burrow beneath the stump, consistent with known adder hibernaculum structures, such as mammal burrows and root systems^7,10^. While direct verification, as in the case of western indigo snakes, was not possible and is in general difficult for hibernacula and this type of search, the probability of a true positive was high. Detection of live adders in their hibernacula should be tested with several trained dogs and ideally with more tagged adders, if possible, to confirm the practical value of this non-invasive method, particularly as dogs may differ individually in their ability to generalize target odors^50^.

### Conclusions and consequences for future practical application

Our study demonstrates, for the first time, that a detection dog can reliably identify adder scent in line-ups at depths of up to 120 cm and in the field at 50 cm under various substrates. To our knowledge, this is also the first successful detection of hibernating adders by a dog. Remarkably, the dog was trained solely with sheds and swabs and not live adders, which is a huge benefit for the animals’ welfare, especially when working with endangered species, in order to keep the disturbance to an absolute minimum during the training process. Although the handler–dog team was relatively inexperienced, potentially increasing the risk of unintentional handler bias during the pilot study, the searches were conducted under the guidance of an experienced wildlife detection dog handler and researcher to ensure methodological reliability. The results of the lineup study suggest that sheds were slightly easier to detect than swabs. When possible, sheds should be favored for training, as they are possible to collect non-invasively and safe without the need to handle a snake, particularly when working with rare or venomous species. However, since the dog was trained with both sample types, it remains unclear whether it would have been able to detect the animals after training with sheds only. This should be tested in the future. Cost-effectiveness plays a key role in the use of detection methods^51^. Wildlife detection dogs can be comparably priced to telemetry studies while being significantly less invasive, at least once the dog is trained^52^. Radiotelemetry only tracks a fraction of a population and is labor-intensive. Compared to visual adder surveys at basking sites, detection dogs can enhance both the quantity and quality of data collected, as they rely on scent rather than visibility^53,54^. This is particularly valuable in dense vegetation where visual surveys are limited^37,55^. A dog can also reduce spatial and gender-specific biases, as female adders emerge later in the season and are less frequently observed at their basking spots near the hibernacula^7,10,56^. While our study showed high sensitivity and efficacy rates for odor samples, this does not guarantee similar success with live adders. Previous studies, such as on indigo snakes, reported a 25% drop in detection performance when using live animals underground^18^. However, performance tends to improve with experience^19^. However, more tagged individuals are needed both during the training phase to generate greater experience in the dog, and in larger-scale studies to accurately assess detection rates during testing. As in other studies of cryptic species, verification remains challenging due to limited effectiveness of tools like endoscopes or wildlife cameras^18,43^. For practical use, guidelines from desert tortoise detection recommend a minimum 75% detection rate and multiple trained teams to validate results. Based on test performance, statistical models can estimate detection probabilities when one or more dogs indicate^43^. This approach could be adapted for adder surveys, ensuring dogs don’t follow each other’s scent. In summary, our findings highlight the potential of detection dogs to locate adder hibernacula and reduce mortality. They also show promise for broader application to other cryptic reptiles and subterranean habitats. However, expanded studies are needed to better quantify effectiveness and environmental influences.

## Electronic Supplementary Material

Below is the link to the electronic supplementary material.

Supplementary Material 1

Supplementary Material 2

## Data Availability

The datasets generated and/or analyzed during the current study are included in the supplementary materials accompanying this article.
