# Supplementary material for: Feasibility study on using a detection dog to localize hibernacula of Vipera berus
Source: Sci Rep. 2026 May 9;16:14681. doi: 10.1038/s41598-026-51778-3 (PMC13156329; doi:10.1038/s41598-026-51778-3)
Supplement: Supplementary file 1 — Supplementary Material 1 [file 41598_2026_51778_MOESM1_ESM.docx]

# Supplementary Information:

Feasibility study on using a detection dog to localize hibernacula of Vipera berus

Madita Schemel^1,2*^, Sascha Buchholz^1,2^, Jelena Mausbach^3^

^1^Institute of Landscape Ecology, University of Münster, Münster, Germany

^2^Centre for Integrative Biodiversity Research and Applied Ecology (CIBRA), University of Münster, Münster, Germany

^3^Artenspürhunde Schweiz, Olten, Switzerland

**Supplementary Table 1:** Overall detection outcomes (correct, false, and missed indications) by depth [cm] and corresponding sensitivity [%] and effectiveness [%].

|  | depth [cm] | correct  indications | missed  indications | false  indications | sensitivity [%] | effectiveness [%] |
| --- | --- | --- | --- | --- | --- | --- |
|  | **0** | 20 | 0 | 0 | 100 | 100 |
|  | **20** | 19 | 1 | 1 | 95 | 90.48 |
|  | **40** | 18 | 2 | 2 | 90 | 81.81 |
|  | **60** | 19 | 1 | 0 | 95 | 95 |
|  | **80** | 20 | 0 | 0 | 100 | 100 |
|  | **100** | 17 | 3 | 2 | 85 | 77.27 |
|  | **120** | 14 | 6 | 2 | 70 | 63.64 |
|  | **Total** | **127** | **13** | **7** | **90.71** | **86.39** |

**Supplementary Table 2:** Number of correct indications, false indications and missed indications by depth [cm] per treatment and corresponding calculated effectiveness [%]

| treatment | depth [cm] | correct  indications | missed  indications | false  indications | effectiveness [%] |
| --- | --- | --- | --- | --- | --- |
| stones/sheds | **0** | 5 | 0 | 0 | 100 |
|  | **20** | 5 | 0 | 0 | 100 |
|  | **40** | 5 | 0 | 0 | 100 |
|  | **60** | 5 | 0 | 0 | 100 |
|  | **80** | 5 | 0 | 0 | 100 |
|  | **100** | 5 | 0 | 0 | 100 |
|  | **120** | 4 | 1 | 1 | 66.66 |
|  | **Total** | **34** | **1** | **1** | **94.44** |
|  |  |  |  |  |  |
| stones/swabs | **0** | 5 | 0 | 0 | 100 |
|  | **20** | 4 | 1 | 1 | 66.66 |
|  | **40** | 5 | 0 | 0 | 100 |
|  | **60** | 4 | 1 | 0 | 80 |
|  | **80** | 5 | 0 | 0 | 100 |
|  | **100** | 5 | 0 | 0 | 100 |
|  | **120** | 4 | 1 | 1 | 66.66 |
|  | **Total** | **32** | **3** | **2** | **86.49** |
|  |  |  |  |  |  |
| peat/sheds | **0** | 5 | 0 | 0 | 100 |
|  | **20** | 5 | 0 | 0 | 100 |
|  | **40** | 4 | 1 | 1 | 66.66 |
|  | **60** | 5 | 0 | 0 | 100 |
|  | **80** | 5 | 0 | 0 | 100 |
|  | **100** | 5 | 0 | 0 | 100 |
|  | **120** | 4 | 1 | 0 | 80 |
|  | **Total** | **33** | **2** | **1** | **91.67** |
|  |  |  |  |  |  |
| peat/swabs | **0** | 5 | 0 | 0 | 100 |
|  | **20** | 5 | 0 | 0 | 100 |
|  | **40** | 4 | 1 | 1 | 66.66 |
|  | **60** | 5 | 0 | 0 | 100 |
|  | **80** | 5 | 0 | 0 | 100 |
|  | **100** | 2 | 3 | 2 | 28.57 |
|  | **120** | 2 | 3 | 0 | 40 |
|  | **Total** | **28** | **7** | **3** | **73.68** |


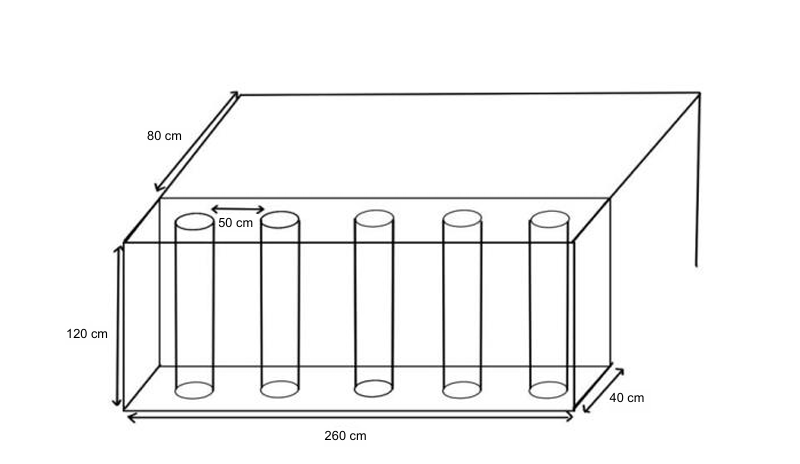


**Supplementary Fig. 1:** The figure shows the depth-adjustable line-up used in the controlled experiments. Five vertically oriented PVC pipes (length 120 cm, diameter 11 cm, spacing 50 cm) were installed inside a box measuring 120 cm in height, 40 cm in width, and 260 cm in length. Substrate was filled into the pipes to predefined heights using an external scale, odor samples were placed at the desired depth, and additional substrate was added above the sample to keep the surface level constant, allowing systematic variation of burial. An additional 80 cm wide platform was attached to the setup, providing a stable and comfortable surface for the dog to walk on and to lie down during odor indication.


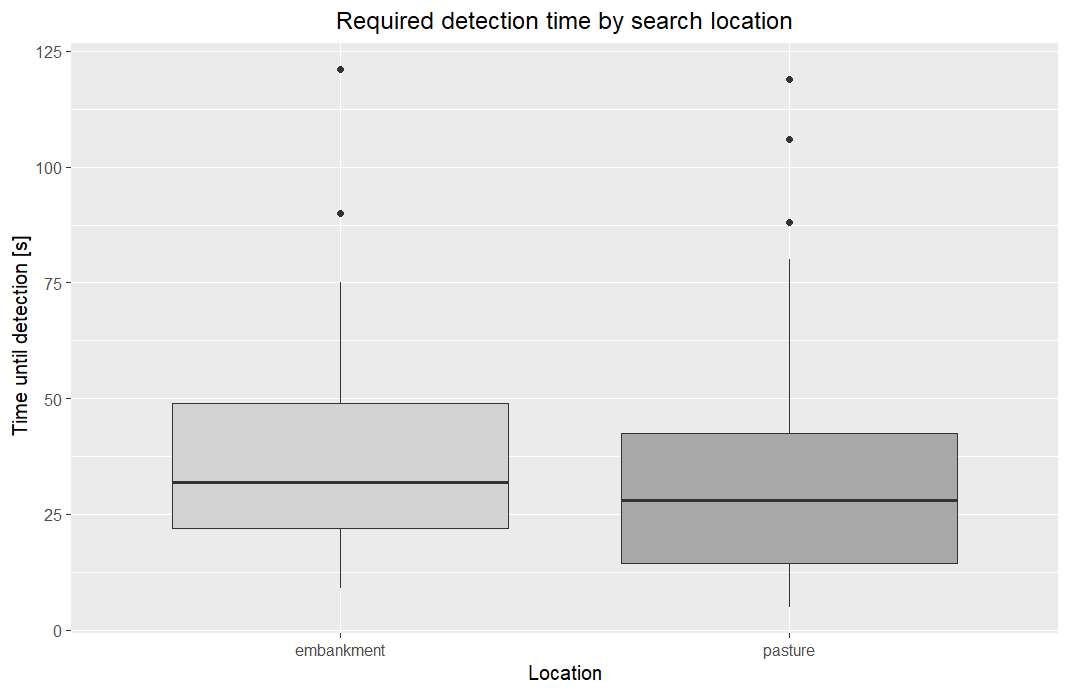


**Supplementary Fig. 2**: Search time required until a positive sample is found [s] depending on the search location
